# Miliary red papules in a newborn: a rare neonatal hemangiomatosis

**DOI:** 10.11604/pamj.2021.38.171.27066

**Published:** 2021-02-16

**Authors:** Nour Mekaoui, Lamya Karboubi

**Affiliations:** 1 Pediatric Medical Emergencies Department, Rabat Children's Hospital, Mohammed V University Rabat, Rabat, Morocco

**Keywords:** Newborn, heamangiomatosis, red papules

## Image in medicine

Hemangiomatosis is the efflorescence of 5 or 6, and up to several hundred, infantile hemangiomas. Several predisposing factors have been isolated: female gender, Caucasian origin, low birth weight and prematurity (these two factors are closely linked and it is above all the birth weight which seems to be the most significant factor). We describe the case of a 15 days old newborn, from a followed up full term pregnancy with a low birth weight at 2200g; admitted for respiratory distress. The examination found more than a hundred angiomas scattered all over the body (A), the head and the mucous membranes (B) whose diameter was less than one centimeter. The examination also showed respiratory distress without hepatomegaly or heart murmur. These cutaneous hemangiomas are often associated with visceral hemangiomas, the most frequent localization of which is the liver. The natural course of these hepatic hemangiomas is parallel to that of skin hemangiomas. They will evolve, like skin forms, towards spontaneous regression, without any treatment. However, they can also; because of intense vascularity, lead to heart failure due to high flow, sometimes severe. A complete clinical examination of the child for signs of heart failure, hepatomegaly, hepatic murmur and the practice of an abdominal ultrasound and doppler. There is no consensus on the number of lesions required to speak of multiple hemangiomas, as well as to provide the indication for a liver ultrasound. Regular monitoring every 2-3 weeks is necessary.

**Figure 1:**
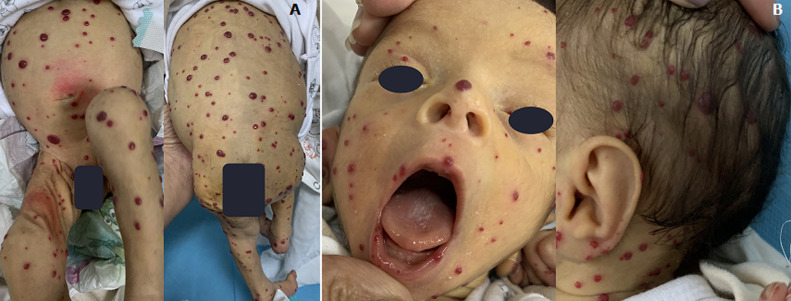
A) miliary angiomas at the body; B) head and mucous membranes

